# Springback Prediction for Pure Moment Bending of Aluminum Alloy Square Tube

**DOI:** 10.3390/ma14143814

**Published:** 2021-07-08

**Authors:** Stanisław Kut, Feliks Stachowicz, Grzegorz Pasowicz

**Affiliations:** 1 Department of Materials Forming and Processing, Rzeszow University of Technology, al. Powstańców Warszawy 8, 35-959 Rzeszów, Poland; stafel@prz.edu.pl; 2 Doctoral School of Engineering and Technical Sciences, Rzeszow University of Technology, al. Powstańców Warszawy 12, 35-959 Rzeszów, Poland; grzegorz.j.pasowicz@lmco.com

**Keywords:** bending, pure moment, springback, square tube

## Abstract

The springback phenomenon occurring during cold forming is the main problem affecting the dimensional accuracy of bent products, especially when bending thin-walled profiles, where there are significant changes in the cross-section geometry. This article presents the results of the analysis of the springback phenomenon occurring during shaping with a pure bending moment of square tubes with the cross-sectional dimensions of 21.5 × 21.5 × 1.8 mm and 25 × 25 × 2.5 mm made of aluminum alloy 6060. The springback characteristics were determined by defining the dependence of the springback coefficient on the set bending radius of the band. The values of the springback coefficient were provided by means of analytical calculations and numerical modeling, which considered changes in the moment of inertia caused by deformation of the cross-section occurring during bending of the pipes. A good agreement of the calculation results with the results of experimental tests was obtained. In addition, the stress state and the state of deformation, as well as the springback characteristics of square-section pipes were compared with the results obtained during bending of a solid bar with the cross-sectional dimensions of 21.5 × 21.5 mm.

## 1. Introduction

During cold plastic forming of bent products, two main phases can be distinguished:

− Active elastic-plastic phase, for which two layers of plasticizing material can be distinguished in the cross-section (subjected to compressive stresses from the inside and tensile stresses from the outside of the bending curvature) and the middle layer of the material in the range of elastic stresses, with a characteristic neutral layer (a layer of zero stresses) and deformations;
− Passive phase (occurring after removing the load with a moment or bending force) during which return elastic deformation takes place, resulting in, among other things, a change of bending curvature and the appearance of residual stresses.

**Figure 1:**
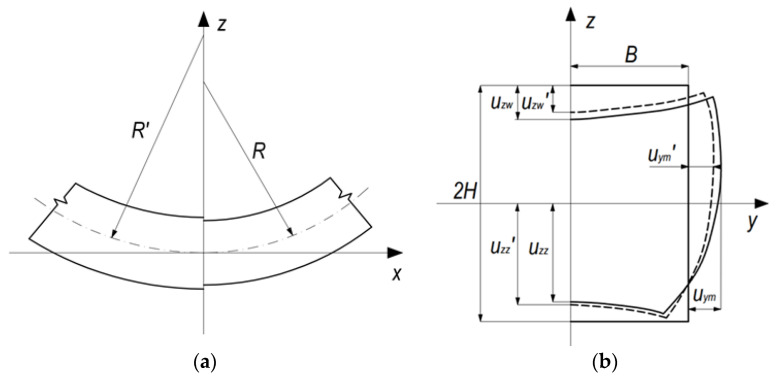
Bending radius (**a**) and characteristic tube dimensions and displacements of the deformed cross section (**b**) in the active bending phase and after unloading (springback).

When applying the theory of elastic-plastic bending to describe the magnitude of the change in the curvature of bending as a result of the occurrence of return elastic deformations, we obtain a generalized relationship [1]:

(1) $$\frac{1}{R}-\frac{1}{R^{\prime }}=\frac{M_{b}}{\mathrm{EI}}$$

where: *R* and *R’*—bending radius in active and passive bending phase, respectively (Figure 1a):

*M_b_*—bending moment;

E—Young’s modulus;

I—moment of inertia of the cross section.

The analysis of the elastic-plastic bending process (in various types of technological processes, as well as for various types of profiles subjected to bending) focused on the qualitative and quantitative description of the springback phenomenon was and still is the subject of many scientific, theoretical and experimental studies. In these studies, different material characteristics determined in tensile or compression tests [2] and changes in the value of Young’s modulus with deformation were considered [3,4]. Various plasticity conditions and different models of strain hardening were considered in the analytical studies [5,6,7]. In the case of bending into large curves, or during bending processes involving axial forces, when analyzing the state of deformation on the cross-section of the bent strip, changes in the position of the neutral layer should be considered [2,8,9]. The value and distribution of residual stresses remaining in the material after unloading depends on the size of the given bending radius and the shape of the bent strip [10,11,12]. When it comes to the analysis of the springback phenomenon during the bending process of tubular profiles, most scientific studies concern pipes with a circular cross-section, while in the studies on pipes with a square (or rectangular) cross-section [12,13,14], the shape of the cross-section after the bending process is not always consistent with the shape of the cross-section observed during our own research [15,16], which may, in a way, result from a different method of imparting bending deformations. When shaping square tubes with pure bending moment, the horizontal walls bend inwards (towards the neutral layer), while the vertical walls bulge outwards. The value of the set bending curvatures depends on the degree of pipe thinness—after exceeding the critical curvature, the cross-section collapses locally [17,18,19].

As expected in the passive phase of the bending process, after removing the load, not only does the curvature of the bent strip change, but the geometry of the cross-section also changes—both the bending curvature and the degree of bending of horizontal and vertical walls (Figure 1). The following indicators were used to describe the shape of the pipe cross-section after bending and after springback (Figure 1b):

$U_{zw}^{\;}$ and $U_{zw}^{\prime }\;$—deflection of the inner horizontal wall, in the active bending phase and after springback, respectively.

$U_{zz}^{\;}$ and $U_{zz}^{\prime }$—deflection of the outer horizontal wall, in the active bending phase and after springback, respectively.

$U_{ym}^{\;}$ and $U_{ym}^{\prime }$—maximum lateral displacement of points of the wall beyond the original cross-section, in the active bending phase and after springback, respectively.

The horizontal walls were those that are normal to the bending plane, the vertical walls were those parallel to the bending plane—"inner” and ”outer” refer, respectively, to walls directed towards, and away from the bending axis.

The purpose of the work is to determine the springback characteristics and changes in the cross-sectional geometry of square tubes extruded from an aluminum alloy, shaped with a pure bending moment. The springback characteristics of the pipe will be compared with the springback characteristics of a solid profile with the same external dimensions of the cross-section. The correctness of the developed numerical model and the determined analytical relationships used to determine changes in the bending curvature and cross-section geometry of square tubes will be verified based on the results of experimental tests.

## 2. Material and Experimental Procedure

The study was carried out on square tubes of 21.5 mm × 21.5 mm × 1.8 mm and 25 mm × 25 mm × 2.5 mm extruded from the aluminum alloy type 6060. The samples cut from the profile wall were tested under uniaxial tensile tension. The Hollomon strain–stress relationship was found with significant reproducibility in the form of $\sigma =322\varepsilon^{0.22}$. Other tensile parameters were as follows: yield stress Rp_0.2_ = 98 MPa; ultimate tensile strength R_m_ = 166 MPa, Young modulus E = 71.0 GPa and Poisson’s ratio ν = 0.33.

Shaping tests under loading with pure bending moment were carried out using a device of our own design [15] mounted on a testing machine. In order to prepare the springback characteristics, the bending tests were carried out sequentially, setting the curvature of the bending in the range κ = 0.5 ÷ 8 m^−1^. At several stages of bending, the bending radius and characteristic indices of the cross-sectional geometry were measured (Figure 1b)—under loading with the bending moment and after removing the load.

## 3. Analytical Modelling of Springback

In our considerations, the value of the springback coefficient K, defined as the ratio of the pipe bending radius under load to the bending radius after removing the load, was adopted as a measure of the value of the return elastic deformations. After transforming the relationship (1), the following was obtained:

(2) $$K=\frac{R}{R^{\prime }}=1-\frac{M_{b}R}{EI}$$

Due to the way the bending process is carried out and the material grade of the bent profiles, the following assumptions were made in the calculations of the springback coefficient:

− The course of the stress–strain relationship for the pipe material describes the same relationship for shaping by stretching and shaping by compression. The value of Young’s modulus is constant, independent of the value and history of deformation.
− Due to the axial symmetry of the cross-section and the relatively small bending curves, it is assumed that the inert layer passes through the center of the bent strip.
− Bending strains obey the principle of volume invariably and the total strain theory.
− The active bending phase is an elastoplastic process, while the unloading phase is an elastic process.

The value of the bending moment determined as the sum of the bending moments of the horizontal walls and vertical walls of the pipe was calculated according to the dependencies presented in [15], considering the changes in their geometry. As the bending curvature increases, the degree of deformation of the cross-section increases, and thus the value of the moment of inertia of cross-section I changes. In order to accurately determine the value of the springback coefficient, it is necessary to calculate the value of the moment of inertia, taking these changes into account. The value of the moment of inertia of the total cross-section is calculated as the sum of the moments of inertia of the horizontal and vertical walls:

(3) $$I=\iint_{S}^{}z^{2}dS=I_{hz}+I_{hw}+2I_{v}$$

The value of the moment of inertia for vertical walls, assuming that their thickness changes only slightly, can be simplified as:

(4) $$I_{v}=4t\int_{0}^{H-t}z^{2}dz=\frac{4}{3}{\left(H-t\right)}^{3}t$$

where *t* is wall thickness.

In turn, the value of the moment of inertia for horizontal walls can be defined as:

(5) $$I_{h}=\iint_{S}^{}{\left(z-U_{Z}\right)}^{2}dS$$

After using the dependencies describing the amount of deflection of these walls Uz after bending [20]:

(6) $$U_{z}=\eta e^{-y^{2}/B^{2}}$$

and the dependence of the η coefficient on the geometric features of the profile and the square of the curvature:

(7) $$\eta =c\frac{B^{3}H}{t}\frac{1}{R^{2}}$$

The value of the constant ***c*** determined by statistical analysis are: ***c_w_*** = 1.57 for inner horizontal walls, and ***c_z_*** = 2.25 for the outer horizontal walls [20].

Thus, the expression describing the value of the moment of inertia for horizontal walls will takes the form:

(8) $$I_{h}=\int_{0}^{B}\int_{0}^{H}(z^{2}-2\eta e^{-\frac{y^{2}}{B^{2}}}+\eta^{2}e^{-\frac{y^{2}}{B^{2}}})dydz$$

Since the directions of strains along the thickness and width of the wall located in the zone of tensile stresses are opposite to the directions of strains along the thickness and width of the wall located in the zone of tensile stresses, the following assumption is made: B and t = const. do not significantly affect the calculation results. After performing the prescribed actions, the formulas for the value of the moment of inertia of the horizontal walls (inner ***I_hw_*** and outer ***I_hz_*** respectively) will take the form:

(9) $$I_{hw}=\frac{2}{3}B\left\{H^{3}-{\left(H-t\right)}^{3}\right\}-2.37\frac{B^{4}H\{H^{2}-(H^{2}-t^{2})\}}{tR^{2}}+4.25\frac{B^{7}H^{2}}{tR^{4}}$$

(10) $$I_{hz}=\frac{2}{3}B\left\{H^{3}-{\left(H-t\right)}^{3}\right\}-3.48\frac{B^{4}H\{H^{2}-(H^{2}-t^{2})\}}{tR^{2}}+9.12\frac{B^{7}H^{2}}{tR^{4}}$$

## 4. Numerical Calculations

FEM modeling of the process of elastic-plastic cold bending of pipes and a solid bar with a square cross-section was carried out using two types of numerical models that considered both geometry and significant nonlinear effects. The first is a full 3D model and the second is a shell model. Both numerical models of the bending process were built based on the experiment, keeping the same geometric parameters of both the bending beam and the bending device. Numerical models have been simplified to a quarter of the experimental model due to the presence of two planes of symmetry along the axis and perpendicular to the cross-section in the middle of the band bent between the supports. Numerical modeling was performed using the commercial MSC.MARC/Mentat 2020 system.This system, is one of the most advanced programs applicable to the modeling of highly nonlinear problems [21]. In both models, the deformed strand of material was defined as a deformable body, and the fixing parts of the bending device as perfectly rigid bodies. The unloading process was initiated by releasing contact and removing the bending moment.

For the discretization of the deformable band in the 3D model, 8-node type 7 cube-shaped elements with dimensions of 0.625 × 0.625 mm for the pipe 25 × 25 × 2.5 mm, and 0.45 × 0.45 mm for the pipe 21.5 × 21.5 × 1.8 mm on the beam cross-section and a length of 1.0 mm were used—which gave about 55,000 finite elements. The size of the elements was selected assuming that the elements on the cross-section were as close to the square as possible and using the relation t/a = 4 where: t—pipe wall thickness, a—finite element size. For the discretization of the deformable band in the shell model, 4-node, quadruple 4 bilinear type 75 elements were used (with dimensions of 0.625 × 1.0 mm for 25 × 25 × 2.5 mm pipe, and 0.45 × 1.0 mm for the pipe 21.5 × 21.5 × 1.8 mm)—which gave approximately 14,000 finite elements. The size shell elements were selected using the relation 2H/a = 40 where: 2H—pipe height (see Figure 1b). The number of integration points in the thickness of the shell element was 11. The remaining simulation parameters for both numerical models were the same.

The isotropic properties of the material were adopted for the calculations because the analyzed profiles are shaped in the process of hot extrusion and are characterized by a homogeneous internal structure. In terms of elastic deformation of the material, the following values of material constants were adopted: Young’s modulus of elasticity E = 71.0 GPa, Poisson’s ratio υ = 0.33. The stress–strain relationship for the material plastic flow range, determined on the basis of the uniaxial tensile test, was introduced to the program in a tabular form. The evolution of the plasticity surface as a result of the strain hardening phenomenon was described using the isotropic model. The calculations used the Huber-Mises–Hencky plasticity condition, the associated Prandtl–Reuss plastic flow law. An implicit scheme for integrating differential equations by the Newton–Raphson method was used.

## 5. Results and Discussion

**Figure 2:**
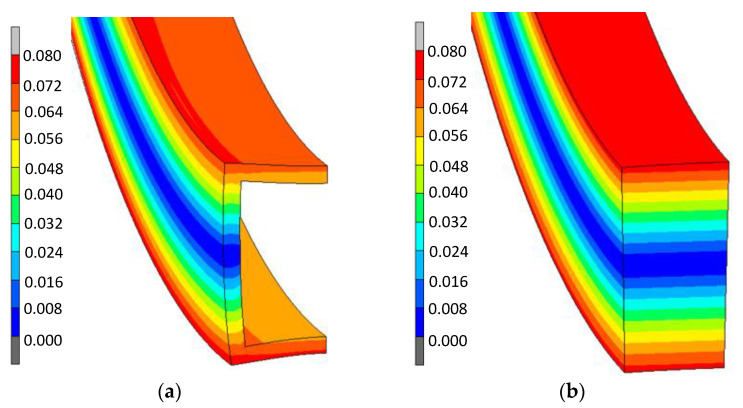
Distribution of equivalent strains on the cross-section of a tube with dimensions of 21.5 × 21.5 × 1.8 mm (**a**) and a bar with dimensions of 21.5 × 21.5 mm (**b**), bent on the radius of the value R = 0.147 m.

**Figure 3:**
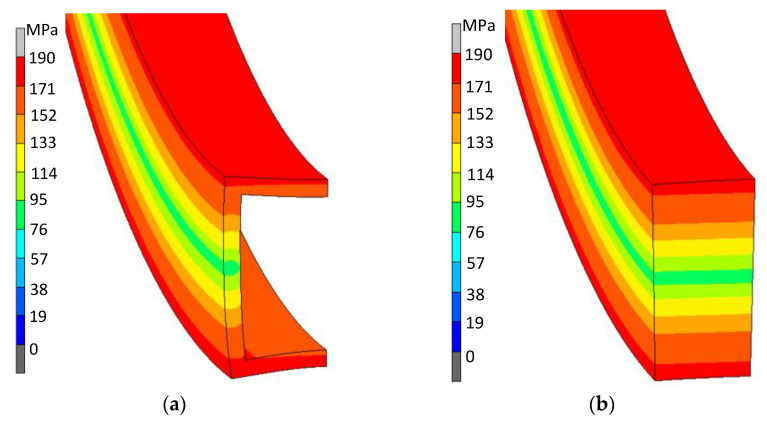
Distribution of Huber–Mises equivalent stresses on the cross-section of a tube with dimensions of 21.5 × 21.5 × 1.8 mm (**a**) and a bar with dimensions of 21.5 × 21.5 mm (**b**), bent on the radius of the value R = 0.147 m.

At the first stage of numerical calculations, the comparison of deformation states and stress states on the cross-section of a pipe and a solid bar with external dimensions of 21.5 × 21.5 mm was made. Based on the distributions of the strain intensity values obtained for the bending radius of the strip with the value R = 1.47 m (Figure 2), it can be seen that both in the case of the pipe and the bar, the strain distribution is symmetrical with respect to the neutral zone in the middle of the cross-section. However, in the case of the pipe, the distribution of strains over the wall thickness, both horizontal and vertical, has the effect of distorting the initial cross-section. A similar observation can be made when comparing the distribution of the stress intensity values on the cross-section of a pipe and a rod (Figure 3).

**Figure 4:**
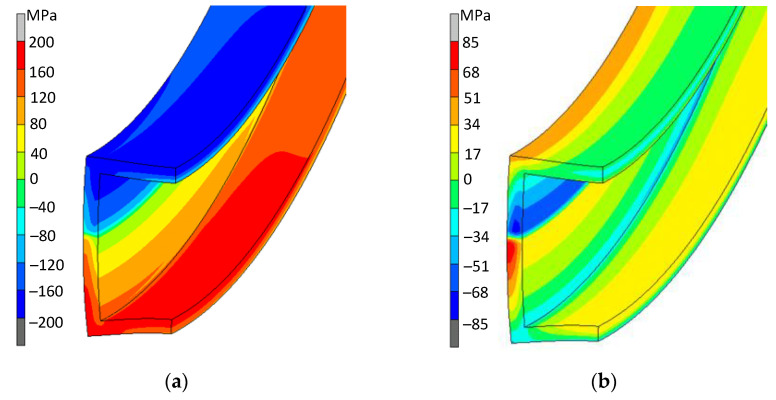
Distribution of axial stresses on the cross-section of a tube with dimensions of 21.5 × 21.5 × 1.8 mm: (**a**) under load, bent to a radius of the value R = 0.138 m, (**b**) after springing (residual stress distribution).

Much greater differences in the stress distributions on the cross-section of the bent strands were observed when considering the stress values along the bar length (in the direction of the Z axis). In the case of bending a solid bar, these distributions were symmetrical with respect to the neutral plane passing through the center of the cross-section—both in the active bending phase (under the action of the bending moment) and after removing the load (springback). In the case of a square tube, the distribution of axial stresses showed a clear influence of the cross-section distortion. Examples of stress distributions are illustrated for a pipe bent on a radius of R = 0.138 m (Figure 4), i.e., for the curvature at which the beginnings of a loss of stability (local pipe collapse) were observed—both in the active bending phase (Figure 4a), as well as after removing the load (Figure 4b). At this stage of bending, the displacement of the inert layer towards the center of the curvature can be observed. The distortion effect of the cross-section of the bent pipe results in a very differentiated distribution of residual stresses—especially in the vertical wall (Figure 4b).

**Figure 5:**
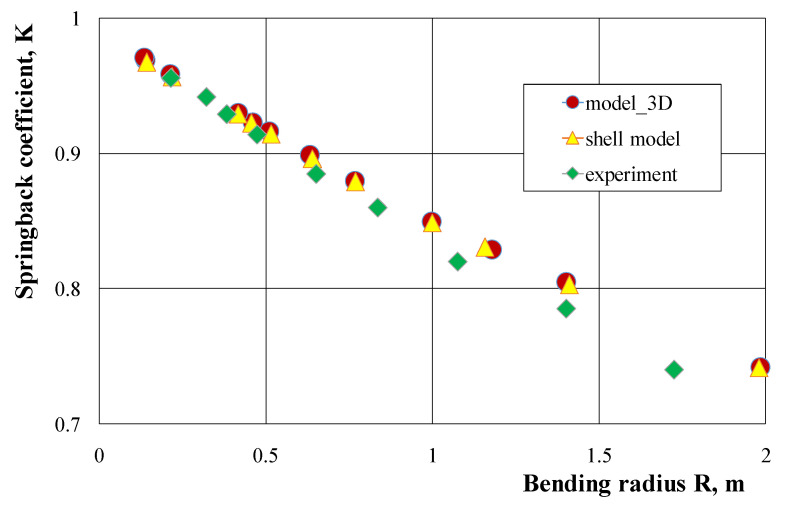
Comparison of the springback characteristics of a square tube with the dimensions of 25 × 25 × 2.5 mm determined on the basis of the results of experimental tests and two FE models.

**Figure 6:**
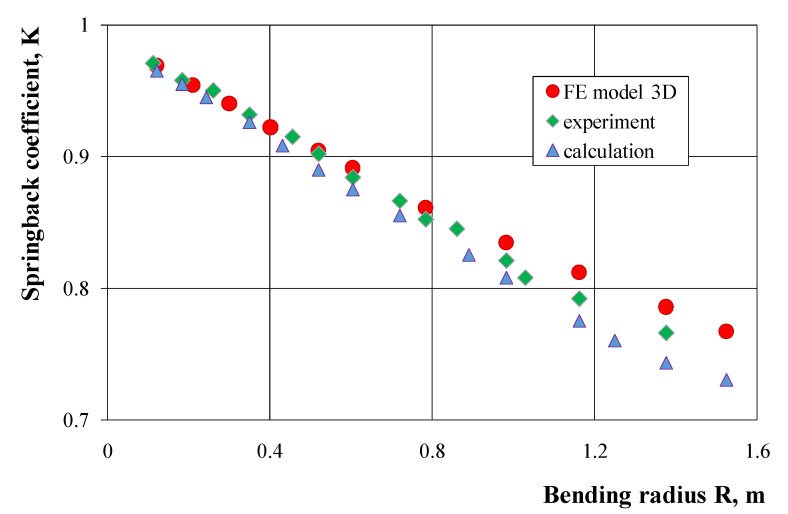
Comparison of the springback characteristics of a square tube with the dimensions of 21.5 × 21.5 × 1.8 mm determined on the basis of the results of experimental tests, analytical calculations and numerical modeling.

**Figure 7:**
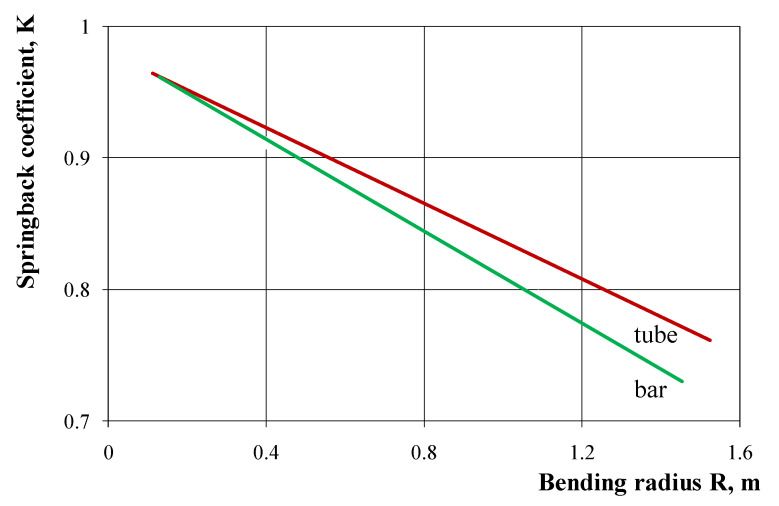
Comparison of the springback characteristics of a square tube with the dimensions of 21.5 × 21.5 × 1.8 mm and a bar with the dimensions of 21.5 × 21.5 mm determined on the basis of numerical modeling.

The results of numerical calculations of springback characteristics carried out with the use of the 3D model and the shell model did not show significant differences (Figure 5); therefore, the shell method may be recommended to determine these characteristics, due to the possibility of using lower computing power and shorter computation time. The results of numerical calculations of the bending characteristics gave slightly overestimated values of the springback coefficient in comparison to the course of these characteristics determined experimentally, both for a pipe with the dimensions of 25 × 25 × 2.5 mm (Figure 5) and with the dimensions of 21.5 × 21.5 × 1.8 mm (Figure 6). On the other hand, the springback characteristics determined by analytical calculations give lower values of the springback coefficient in comparison to the results of experimental measurements (Figure 6). The greatest differences in the springback coefficient values obtained on the basis of experimental tests, and those calculated analytically or numerically, occur for small bending curves. As the curvature of the bending increases, these differences decrease and for the largest applied bending curves, the values of the springback coefficient are the same, close to the value of the springback coefficient K = 1.0 (no occurrence of return elastic deformations). The results of numerical calculations (with the assumptions made) for a pipe and a solid bar made of the 6060 aluminum alloy obtained in the extrusion process show a linear dependence of the springback coefficient value on the bending radius of the strip (Figure 7). For small bending curves, the values of the springback coefficient away from the solid bar are clearly lower than the values determined for the pipe with the same external cross-section dimensions. These differences decrease as the curvature of the bend increases and are almost the same for small curvatures.

As mentioned earlier, after the bending moment re-moving, we observe elastic deformations, as a result of which the strip curvature and the degree of cross-section deformation change, while after springback the bending radius increases, and the deflection of the walls (both horizontal and vertical) decreases. The results of the calculations carried out for the three selected bending radius values showed that for both analyzed types of pipes the highest displacement value applies to the horizontal wall ***U_zz_*** outside the bending curvature, and the smallest deflection value is observed for the vertical wall ***U_ym_*** (Table 1). To compare the value of the elastic deformation of the pipe walls with the value of the springback coefficient of the entire strip, the pipe wall springback index was introduced, defined as:

(11) $$K_{w}=\frac{U^{\prime }}{U}$$

where: *U* and *U′* is the value of the selected wall deflection index, respectively, under load and after springback.

The results of calculations of the springback index defined in this way (Table 2) showed that for both types of pipes the values of the springback index of the vertical walls have the highest value, close to the value of the springback coefficient of the entire strip. The lowest values of the springback index were found for the horizontal inner wall. This is due to the fact that near the center of the vertical walls there is a layer in which elastic stresses occur in the active phase of the process, while the horizontal walls are in the range of plasticizing stress. On the other hand, the values of the springback index lower for the internal horizontal wall than for the external horizontal wall result from the fact that the thickness of the internal wall increases due to the impact of compressive stresses (which increases its stiffness), while the tensile stress acts in the external wall, resulting in its reduction thickness (thereby reducing its stiffness).

## 6. Conclusions

This work presents the results of the analysis of the springback phenomenon observed during elastic-plastic cold shaping of profiles loaded with a pure bending moment, with particular emphasis on changes in the cross-sectional geometry of square tubes bent. The results of analytical calculations and numerical modeling were in good agreement with the results of experimental tests. The results of numerical modeling of bending pipes with a square cross-section were compared with the results of bending a solid bar. The main conclusions of this work can be summarized as follows:

Analytical calculations of the springback coefficient (considering changes in the geometry of the cross-section, resulting in a change in the value of the moment of inertia) gave results that were lower in comparison to the results of the experimental tests. In the case of numerical modeling, the results were overestimated. These differences were greater the greater the bending radius (less curvature). For the bending radius R = 1.37 m, these differences were—3.0% and 2.5% respectively, for analytical calculations and numerical modeling.

− No significant differences were found in the calculation results of the springback coefficient for the discretization of the deformable band in the 3D model or in the shell model;therefore, the shell method may be recommended to determine these characteristics, due to the possibility of using lower computing power and shorter calculation time.
− Distribution of the intensity of strains and stresses on the cross-section of the bent strand showed symmetry with respect to the neutral layer, especially in the case of bending a solid bar. In the case of bending pipes, these distributions are more diversified as a result of overlapping components of strains and stresses resulting from bending of the pipe walls.
− With a high bending curvature, at which the location of deformations leading to a local collapse of the pipe occurs, a shift of the neutral layer towards the center of the curvature was found—both in the active bending phase and after springback.
− Comparison of the springback characteristics of the pipes and the rod indicates a greater intensity of the springback of the full profile, especially for small bending curves.
− The value of the elastic deformation index of the vertical pipe walls is comparable to the springback value of the entire profile. The lowest value of the springback index was found for the horizontal inner wall, which results from the effect of compressive stresses resulting in an increase in its thickness, and thus an increase in its stiffness.

## Figures and Tables

**Table 1:** Dependence of the springback coefficient value and wall deflection indices value on the bending radius of the 25 × 25 × 2.5 and 21.5 × 21.5 × 1.8 profile, before and after unloading.

Profile Type	Bending Radius, R, m	Springback Coefficient K	The Values of the Deflection Indexes of the Walls Under Load, mm			The Values of the Deflection Indexes of the Walls after Unloading, mm		
			U_wz_	U_zz_	U_ym_	U’_wz_	U’_zz_	U’_ym_
25 × 25 × 2.5	0.556	0.916	0.084	0.144	0.124	0.065	0.125	0.112
	0.223	0.958	0.209	0.559	0.322	0.177	0.514	0.303
	0.142	0.969	0.663	1.219	0.551	0.584	1.145	0.545
21.5 × 21.5 × 1.8	0.526	0.906	0.014	0.119	0.095	0.010	0.101	0.087
	0.224	0.949	0.226	0.469	0.237	0.182	0.423	0.223
	0.147	0.964	0.610	1.001	0.444	0.535	0.929	0.427

**Table 2:** The values of the walls springback index of the 25 × 25 × 2.5 and 21.5 × 21.5 × 1.8 profile.

Profile Type	Bending Radius R, m	Springback Coefficient K	The Values of the Springback Index of the Profile Walls, K_w_		
			K_wz_	K_zz_	K_ym_
25 × 25 × 2.5	0.556	0.916	0.774	0.868	0.903
	0.223	0.958	0.846	0.919	0.931
	0.142	0.969	0.881	0.939	0.959
21.5 × 21.5 × 1.8	0.526	0.906	0.721	0.848	0.916
	0.224	0.949	0.805	0.902	0.941
	0.147	0.964	0.877	0.928	0.962

## Data Availability

Not applicable.
